# Polycomb-mediated silencing in neuroendocrine prostate cancer

**DOI:** 10.1186/s13148-015-0074-4

**Published:** 2015-04-03

**Authors:** Pier-Luc Clermont, Dong Lin, Francesco Crea, Rebecca Wu, Hui Xue, Yuwei Wang, Kelsie L Thu, Wan L Lam, Colin C Collins, Yuzhuo Wang, Cheryl D Helgason

**Affiliations:** Department of Experimental Therapeutics, British Columbia Cancer Research Centre, 675 W 10th Avenue, Vancouver, BC V5Z 1 L3 Canada; Interdisciplinary Oncology Program, Faculty of Medicine, University of British Columbia, 675 W 10th Avenue, Vancouver, BC V5Z 1 L3 Canada; Vancouver Prostate Centre, 899 West 12th Avenue, Vancouver, BC V5Z 1 M9 Canada; Department of Urologic Sciences, Faculty of Medicine, University of British Columbia, 2775 Laurel Street, Vancouver, BC V5Z 1 M9 Canada; Department of Integrative Oncology, Genetics Unit, British Columbia Cancer Research Centre, 675 W 10th Avenue, Vancouver, BC V5Z 1 L3 Canada; Department of Surgery, University of British Columbia, 910 W 10th Avenue, Vancouver, BC V5Z 4E3 Canada

**Keywords:** Neuroendocrine prostate cancer, Patient-derived xenografts, Epigenetics, Polycomb, CBX2, EZH2, Therapeutics, Prognosis

## Abstract

**Background:**

Neuroendocrine prostate cancer (NEPC) is a highly aggressive subtype of prostate cancer (PCa) for which the median survival remains less than a year. Current treatments are only palliative in nature, and the lack of suitable pre-clinical models has hampered previous efforts to develop novel therapeutic strategies. Addressing this need, we have recently established the first *in vivo* model of complete neuroendocrine transdifferentiation using patient-derived xenografts. Few genetic differences were observed between parental PCa and relapsed NEPC, suggesting that NEPC likely results from alterations that are epigenetic in nature. Thus, we sought to identify targetable epigenetic regulators whose expression was elevated in NEPC using genome-wide profiling of patient-derived xenografts and clinical samples.

**Results:**

Our data indicate that multiple members of the polycomb group (PcG) family of transcriptional repressors were selectively upregulated in NEPC. Notably, CBX2 and EZH2 were consistently the most highly overexpressed epigenetic regulators across multiple datasets from clinical and xenograft tumor tissues. Given the striking upregulation of PcG genes and other transcriptional repressors, we derived a 185-gene list termed ‘neuroendocrine-associated repression signature’ (NEARS) by overlapping transcripts downregulated across multiple *in vivo* NEPC models. In line with the striking upregulation of PcG family members, NEARS was preferentially enriched with PcG target genes, suggesting a driving role for PcG silencing in NEPC. Importantly, NEARS was significantly associated with high-grade tumors, metastatic progression, and poor outcome in multiple clinical datasets, consistent with extensive literature linking PcG genes and aggressive disease progression.

**Conclusions:**

We have explored the epigenetic landscape of NEPC and provided evidence of increased PcG-mediated silencing associated with aberrant transcriptional regulation of key differentiation genes. Our results position CBX2 and EZH2 as potential therapeutic targets in NEPC, providing opportunities to explore novel strategies aimed at reversing epigenetic alterations driving this lethal disease.

**Electronic supplementary material:**

The online version of this article (doi:10.1186/s13148-015-0074-4) contains supplementary material, which is available to authorized users.

## Background

With a median survival of less than a year, neuroendocrine prostate cancer (NEPC) represents the most aggressive prostate malignancy and only a small fraction of NEPC patients benefit from current treatments [1]. While NEPC may arise *de novo*, most cases result from the transdifferentiation of a typical prostate adenocarcinoma (PCa) into NEPC following androgen-deprivation therapy (ADT) [2,3]. Histologically, NEPC is characterized by the presence of small round cells with a prominent nucleus and scant cytoplasm. Typically arranged in a monomorphic pattern, NEPC cells stain positive for neuroendocrine markers such as chromogranin A (CHGA) and synaptophysin (SYP) but negative for PCa markers like androgen receptor (AR) and prostate-specific antigen (PSA) [4]. Since NEPC cells lack AR expression, they probably arise by positive selection following AR suppression, thus providing an adaptive mechanism to achieve castration resistance [5]. Accordingly, due to the recent FDA approval of potent AR-targeting drugs in patients receiving ADT, NEPC incidence is expected to dramatically rise in the near future, creating an urgent need for improved therapeutics [5]. Emerging evidence suggests that epigenetic alterations may be involved in neuroendocrine transdifferentiation (NETD) [6,7], providing unexplored opportunities to identify novel drug targets for this invariably lethal disease.

Epigenetics is a broad term that encompasses all mitotically and meiotically heritable changes in chromatin structure and gene expression that do not result from alterations in DNA sequence [8]. Epigenetic regulation is conferred by covalent modifications on DNA and histones, which define the transcriptional activity of surrounding genomic regions [9]. Historically, the first epigenetic modification discovered was DNA methylation at CpG dinucleotides, a mark typically correlating with transcriptional silencing [10,11]. On histones, numerous chemical modifications can be enzymatically added and removed from N-terminal tails by a large network of epigenetic regulators (EpRs), giving rise to a highly complex ‘histone code’ [12]. Examples of post-translational modifications include methylation, acetylation, ubiquitination, phosphorylation, and many others that are starting to gain recognition [13]. The transcriptional effect conferred by these modifications depends on the particular chemical mark as well as the residue on which it is deposited [14]. Three main types of EpRs dynamically control chromatin: 1) writers, which catalyze the addition of covalent modifications; 2) erasers, which remove these marks; and 3) readers, which directly bind epigenetic modifications [15]. Readers can simultaneously interact with chromatin-remodeling factors, thereby reshaping the epigenome in a reversible fashion [15]. Importantly, almost all EpRs lie downstream of signal transduction pathways, allowing for dynamic chromatin modulation in response to external cues [16]. Accordingly, epigenetic regulation plays a key role in fundamental processes that involve changes in phenotypic identity such as development and cancer [17,18]. Since epigenetic modifications are reversible, EpRs can be pharmacologically targeted by small molecule inhibitors, and a rapidly growing number of ‘epi-drugs’ are receiving FDA approval [19].

Given the growing interest in identifying clinically relevant epigenetic alterations, considerable attention has been given to the polycomb group (PcG) gene family in the context of human cancer [20]. PcG proteins represent important epigenetic silencers that have been strongly linked to cellular de-differentiation and malignant progression [21]. Assembling into two main polycomb repressive complexes (PRC1 and PRC2), these proteins regulate hundreds of genes involved in major cell fate decisions [22]. In the classical PcG silencing model, PRC2 trimethylates histone H3 at lysine 27 (H3K27me3), through its catalytic subunit EZH2 [23]. This repressive chromatin mark can be directly recognized by the N-terminal chromodomain of chromobox proteins (CBX2,4,6,7,8) [24], which then recruit PRC1 members to chromatin via a C-terminal domain [25]. At genomic sites, PRC1 can then monoubiquitylate histone H2A (H2AK119ub) through its catalytic components RING1A or RING1B, which further represses PcG target *loci* [26]. To date, dysregulation of PcG-mediated silencing has been observed in many aggressive tumor types but has not been studied in NEPC. Interestingly, PcG genes are required for neurogenesis and neural stem cell survival [27-29], implying that they may regulate differentiation into neuronal lineages. In line with this idea, we and others have recently shown that EZH2 mRNA levels are upregulated in NEPC [7], suggesting that alterations in PcG-mediated repression may be involved in NEPC pathogenesis.

Given the lack of xenograft and cell line models to study NEPC, we established the first *in vivo* model of ADT-induced NEPC using patient-derived xenografts implanted in the mouse subrenal capsule at the Living Tumor Laboratory [6]. Our initial analysis revealed that the original PCa (LTL331) and the relapsed NEPC (LTL331R) tumor lines share a remarkably similar genetic profile, suggesting that epigenetic alterations were likely to drive NEPC [6]. We therefore conducted comparative gene expression analysis between LTL331R and LTL331, as well as in a clinical NEPC cohort, to identify EpRs that were differentially expressed in NEPC. Our data demonstrate that multiple PcG family members are overexpressed in NEPC, notably CBX2 and EZH2. Consistent with these results, we derived a neuroendocrine-associated repression signature (NEARS) that predicted aggressive disease progression and was enriched in PcG targets. Overall, our results support a clinically relevant function for PcG-mediated silencing, revealing novel targets for development of epigenetic therapies in the context of lethal NEPC.

## Results

### Expression profiling of epigenetic regulators in NEPC

To uncover potential therapeutic targets in NEPC, we set out to identify upregulated genes in the LTL331R/LTL331 xenograft model, as well as in a clinical NEPC dataset containing gene expression profiling of PCa and NEPC patient tumors [7]. We initially established a list of EpRs using criteria that would maximize the translational application of identified targets. For these reasons, we restricted our list to the epigenetic writers, erasers, and readers regulating histone acetylation and methylation, as well as DNA methylation [30]. Furthermore, the selected genes were also functionally classified into those associated with transcriptional activation or repression, and EpRs for which the transcriptional role remains unclear. Using a panel of recent comprehensive reviews, we derived a list of 147 EpRs that we subsequently analyzed in our NEPC expression datasets (Table 1, Additional file 1: Table S1).

**Table 1:** **Distribution of 147 investigated epigenetic regulators across different epigenetic modifications, activities, and transcriptional effects**

**Criteria**	**Epigenetic regulator distribution**		
Epigenetic modification	DNA methylation	Histone acetylation	Histone methylation
	13 (9%)	65 (44%)	69 (47%)
	Writer	Eraser	Reader
	53 (36%)	40 (27%)	54 (37%)
	Activation	Repression	Unclear
	72 (49%)	66 (45%)	9 (6%)
Total	147 genes		

**Figure 1:**
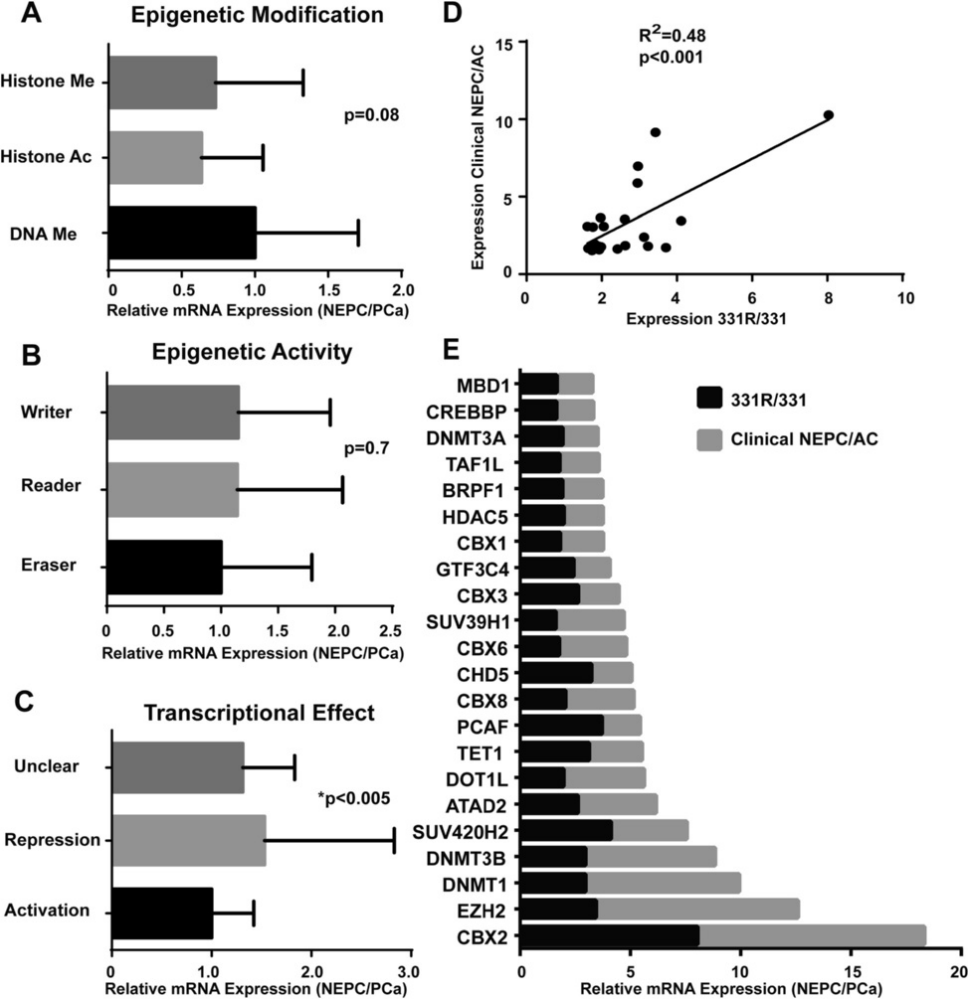
**Differential expression of epigenetic regulators in NEPC.** Average fold change of individual EpRs grouped by distinct **(A)** epigenetic modifications, **(B)** epigenetic activities, and **(C)** transcriptional effects in clinical NEPC/PCa and the 331R/331 model. **(D)** Expression correlation of 22 EpRs upregulated by more than 1.5-fold in clinical NEPC/PCa and the 331R/331 model. **(E)** Expression levels of individual upregulated EpRs in clinical NEPC/PCa and the 331R/331 model.

To investigate the epigenetic landscape of NEPC, we assessed the differential expression of our EpR list in the clinical NEPC cohort and the LTL331R/LTL331 microarray dataset [6,7]. First, we determined if there was preferential upregulation of readers, writers, or erasers and observed no significant difference (Figure 1A, non-significant, Kruskal-Wallis test). The same analysis was conducted investigating factors affecting different chromatin modifications (histone acetylation and methylation, DNA methylation) and demonstrated that no expression differences could be detected between EpRs associated with these chemical marks (Figure 1B, non-significant, Kruskal-Wallis test). However, we found that the mRNA levels of transcriptional repressors were significantly higher than that of activators in NEPC, which may result in a more repressed chromatin state that potentially regulates neuroendocrine differentiation (Figure 1C, *P* < 0.05, Kruskal-Wallis test). Next, starting from our 147 EpR list described earlier (Table 1), we selected 22 genes that were upregulated in both the LTL331R/LTL331 model and the clinical cohort (expression shown in Additional file 1: Table S1, cutoff fold change (FC) in both >1.5). Expression of these 22 EpRs was significantly correlated between the clinical cohort and the LTL331R model (Figure 1D, *R*^2^ = 0.48, *P* < 0.001, Spearman test), indicating that gene expression in our xenograft model accurately reproduced the transcriptional profiles observed in the clinical setting. As expected, most of the selected EpRs were preferentially involved in transcriptional repression, accounting for 68% of all selected EpRs (Figure 1E). To assess the clinical relevance of these genes, we investigated whether their elevated expression was associated with specific parameters of prostate cancer progression in the Oncomine database [31]. Using stringent inclusion criteria (*P* < 0.005, odds/ratio > 5, top 10% overexpressed), we identified five independent Oncomine studies in which this gene list was significantly upregulated in disseminated prostate tumors (Additional file 2: Table S2). Since these datasets were mostly derived from localized PCa tissue, this indicates that elevated expression of those 22 EpRs in primary PCa may predispose tumors for an aggressive and metastatic progression, consistent with NEPC pathogenesis.

To further characterize these epigenetic alterations, we focused on individual genes that were aberrantly regulated in both the clinical NEPC cohort and in LTL331R. An important finding was that the PcG H3K27me3 reader CBX2 was the most highly overexpressed transcript in both datasets (Figure 1E, FC 331R/331 = 8.2, FC NEPC/PCa = 10.2). Interestingly, the H3K27me3 writer EZH2 was the second most highly upregulated transcript (Figure 1E, FC 331R/331 = 3.4, FC NEPC/PCa = 9.2), implying that H3K27me3 and its downstream epigenetic effects may be potentiated in the molecular context of NEPC. Of note, the selected gene list also included two other PRC1-containing CBX proteins, CBX6 and CBX8 (Figure 1E), further supporting a role for dysregulated PcG-mediated silencing during neuroendocrine transdifferentiation. In addition to the increase in PcG genes themselves, we also observed that 73% of non-PcG repressors in our list of upregulated EpRs have been reported to directly interact with at least one PcG member (Additional file 3: Table S3). These PcG-interacting proteins were mainly involved in DNA methylation (DNMT1, DNMT3A, DNMT3B, MBD1) and histone methylation (SUV39H1, DOT1L, CHD5, CBX3), suggesting that the upregulation of other EpRs may contribute to the effect of altered PcG-mediated silencing in NEPC [32,33].

### PcG gene expression in LTL patient-derived xenografts

**Figure 2:**
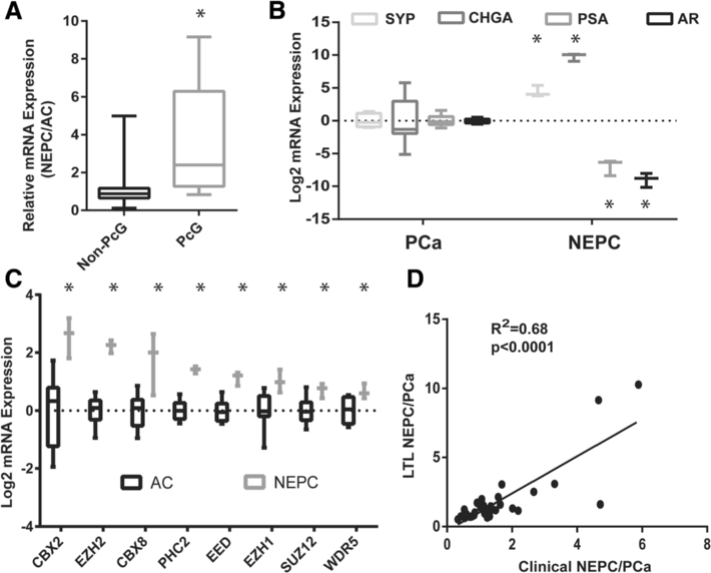
**Coordinated increase in PcG gene expression. (A)** Average fold change of non-PcG and PcG genes from unselected 147 EpR list and selected 22 EpR list. **(B)** Expression of typical prostate neuroendocrine (SYP, CHGA) and adenocarcinoma (AR, PSA) markers in selected xenograft models. **(C)** Coordinated upregulation of core PRC1 and PRC2 members led by CBX2 and EZH2 in selected xenograft models. **(D)** Significant correlation between PcG gene expression in all LTL xenograft models and 331R/331 model.

Since the most aberrantly expressed transcripts were members of the PcG family (Figure 2A, *P* < 0.001, Mann-Whitney *U* test), we compared the expression of 36 known PcG genes (Additional file 4: Table S4) in ten PCa and three NEPC xenografts from the Living Tumor Laboratory using microarray data we have previously published [6] (models and their properties listed in Additional file 5: Table S5). To ensure that our xenograft models retained typical features of their respective subtype, we assessed expression of molecular markers specific to PCa (AR, PSA) and NEPC (SYP, CHGA) in the investigated Living Tumor Lab (LTL) tumor lines. As expected, expression of these markers segregated perfectly between the two malignancies. AR and PSA were selectively upregulated 489- and 124-fold in PCa over NEPC, respectively (Figure 2B, *P* < 0.0001, Mann-Whitney *U* test). In contrast, SYP and CHGA respectively exhibited a 21- and 854-fold enrichment in neuroendocrine tumor lines compared to PCa models (Figure 2B, *P* < 0.0001, Mann-Whitney *U* test). Having confirmed that our patient-derived xenografts were transcriptionally representative of each subtype, we assessed the expression of PcG genes in the same models. Of the 36 PcG genes that were queried (Additional file 4: Table S4), ten were significantly upregulated (Figure 2C, *P* < 0.05, Mann-Whitney *U* test). As observed in the LTL331R/LTL331 model and in the clinical NEPC dataset, CBX2 and EZH2 were again the two most highly overexpressed genes with fold changes of 5.8 and 4.7, respectively (Figure 2C, *P* < 0.0001, Mann-Whitney *U* test). In addition, overabundance of CBX6 and CBX8 transcripts was also observed in neuroendocrine tumor lines (Figure 2C, *P* < 0.05, Mann-Whitney *U* test), consistent with our previous findings. Notably, all core PRC2 members (EED, EZH1, EZH2, SUZ12) were significantly upregulated in NEPC (Figure 2C, *P* < 0.05, Mann-Whitney *U* test). In addition, we found a significant correlation between expression of PcG genes in the LTL331R/LTL331 model and in the clinical cohort (Figure 2D, *R*^2^ = 0.68, *P* < 0.0001, Spearman test), validating reproducible PcG upregulation across all investigated datasets.

**Figure 3:**
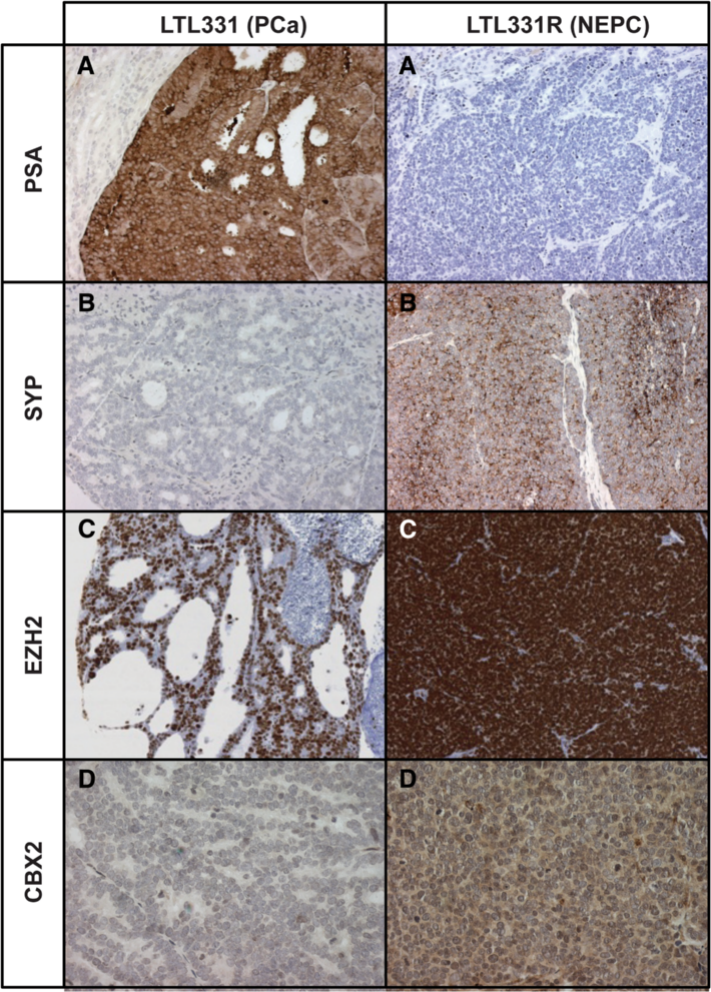
**CBX2 and EZH2 protein expression in NEPC.** Immunohistochemical analysis of **(A)** PSA, **(B)** SYP, **(C)** EZH2, and **(D)** CBX2 in the 331R/331 xenograft model (×20).

Since we found that CBX2 and EZH2 were consistently the most upregulated EpRs in our NEPC models, we further investigated the molecular profiles of these two PcG members. Using immunohistochemistry (IHC), we analyzed CBX2 and EZH2 protein expression in the LTL331R/LTL331 model. We first used antibodies to PSA and SYP to confirm that LTL331 and LTL331R retain the histological features of PCa and NEPC, respectively (Figure 3). As expected, PSA expression was very strong in LTL331 while LTL331R displayed undetectable levels. In contrast, SYP immunoreactivity was strictly restricted to LTL331R, in line with the expected histological profiles of each tumor line. Having validated our positive and negative controls for each subtype, we analyzed CBX2 and EZH2 protein levels in both tumor lines (Figure 3). In accordance with high mRNA levels, CBX2 exhibited strong immunoreactivity in LTL331R while displaying only weak positivity in LTL331. Similarly, EZH2 protein expression was extremely high in basically all LTL331R cells, although most LTL331 cells also displayed moderate to strong EZH2 immunostaining. Taken together, these data demonstrate that CBX2 and EZH2 are highly overexpressed at both the mRNA and protein levels in NEPC.

**Figure 4:**
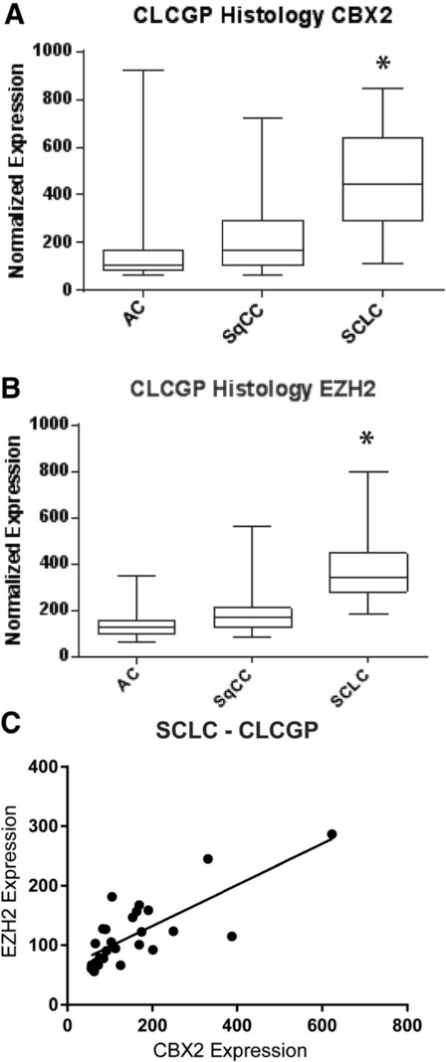
**Regulation of PcG proteins CBX2 and EZH2 in lung cancer subtypes. (A)** CBX2 and **(B)** EZH2 mRNA levels in non-neuroendocrine (AC and SqCC) and neuroendocrine (SCLC) lung malignancies. **(C)** Correlation between CBX2 and EZH2 mRNA levels in SCLC (data from CLGCP).

Focusing on EZH2 and CBX2, we investigated whether elevated expression of these two PcG members also occurred in small cell lung cancer (SCLC), since it represents a neuroendocrine malignancy that closely resembles NEPC histologically and molecularly [34,35]. The relative mRNA levels of CBX2 and EZH2 were therefore assessed in SCLC and compared to two epithelial lung cancer subtypes, lung adenocarcinoma (AC) and squamous cell carcinoma (SqCC) [36]. We report that both CBX2 and EZH2 were significantly overexpressed in SCLC compared to epithelial counterparts [37] (Figure 4A,B, *P* < 0.0001 for both genes, Kruskall-Wallis test). In addition, CBX2 and EZH2 expression was strongly correlated in a clinical SCLC cohort (*R*^2^ = 0.59, Figure 4C), suggesting that these two PcG proteins likely act in concert. Thus, the selective involvement of CBX2 and EZH2 in aggressive neuroendocrine tumors may not be restricted to NEPC.

Another similarity between NEPC and SCLC is that both malignancies were reported to have frequent RB1 inactivation, which contributes to their high proliferative rate [38,39]. Thus, we investigated the RB1 status in our patient-derived xenograft models. As expected, RB1 expression was relatively high in almost all PCa xenografts while the expression of CBX2 and EZH2 was considerably lower (Additional file 6: Figure S1). Conversely, the expression of RB1 was undetectable in the NEPC tumor lines LTL352 and LTL370 while LTL331R exhibited a modest increase (Additional file 6: Figure S1). Genomic characterization of these models revealed that both LTL352 and LTL had homozygous RB1 deletion while LTL331R had a monoallelic loss and a hemyzygous mutation in RB1 (Wyatt *et al*. unpublished). Taken together, these data suggest that aberrant PcG-mediated silencing may cooperate with RB1 inactivation to sustain the high proliferative rate of NEPC.

### Polycomb silencing and neuroendocrine-associated repression signature

**Figure 5:**
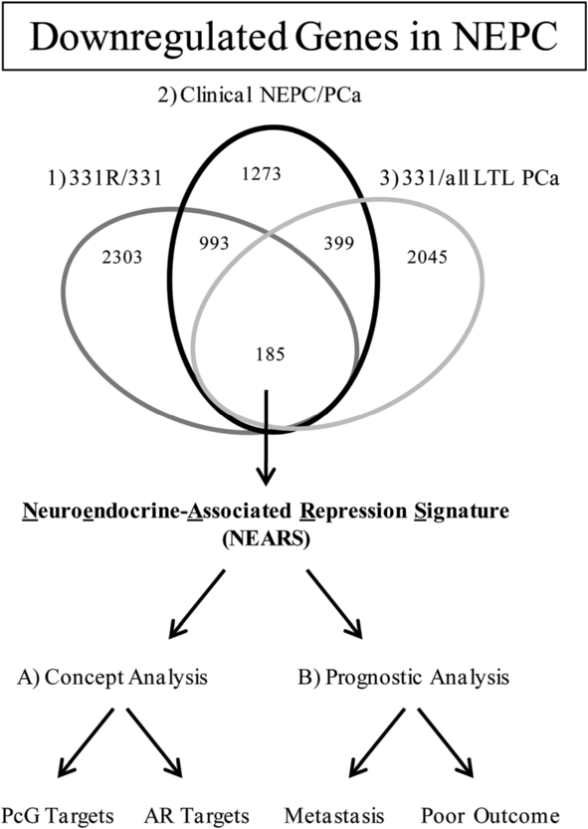
**Establishment and Oncomine analysis of a 185-gene ‘neuroendocrine-associated repression signature’ (NEARS) derived from datasets originating from NEPC models.**

Since our initial analysis revealed that epigenetic repressors, in particular the PcG genes, were significantly upregulated in NEPC, we investigated whether we could infer molecular and clinical information from the genes downregulated in NEPC. We therefore derived a gene signature by combining the list of genes whose expression decreased by at least twofold (50%) in three independent scenarios: 1) LTL331R *vs* LTL331, 2) clinical NEPC *vs* PCa, and 3) LTL331 *vs* all other LTL PCa. The latter was investigated under the hypothesis that LTL331 may be ‘predisposed’ to transdifferentiate compared to other PCa tumor lines. Thus, using this 50% threshold, we established a list of 185 genes silenced in NEPC, which we termed the NEARS (Figure 5, Additional file 7: Table S6). We first used the Oncomine database [31] to identify molecular ‘concepts’ (that is, sets of genes derived from previously published experiments) that were significantly associated with NEARS. In line with the striking upregulation of PcG genes, this analysis revealed that, of the thousands of concepts present on Oncomine, 6 of the top 12 concepts were directly linked to PcG-mediated silencing (Table 2, odds ratios = 3.7 to 5.0, *P* = 3.4 × 10^−9^ to 1.2 × 10^−15^). These six concepts specifically overlapped with target genes of known PcG members CBX8, SUZ12, and EED, as well as H3K27me3. Of note, these concepts were derived from experiments conducted either in embryonic stem cells or in embryonic fibroblasts, consistent with the role of PcG complexes in undifferentiated cells [22]. As an unexpected indicator of quality control, we also found that two concepts strongly linked to NEARS included ‘downregulated genes in prostate cancer after androgen ablation therapy’ and ‘upregulated genes in prostate cancer cells in response to synthetic androgen R1881’, two concepts sharing 19 genes (odds ratios = 20.5, *P* < 3.662 × 10^−18^). These concepts describe genes that are likely AR-regulated [40], and therefore their downregulation is expected in NEPC cells given their lack of AR expression. Taken together, our results demonstrate that NEARS is enriched in PcG targets and preferentially contains genes regulated by AR transactivation.

**Table 2:** **List of top 12 literature-derived concepts most significantly associated with NEARS**

**NEARS literature-defined Oncomine concept analysis**					
**Rank**	**Concept**	**PcG-related**	***P*** **value**	***Q*** **value**	**Odds ratio**
1	CBX8 target genes in human embryonic fibroblasts	X	1.2E-15	9.6E-12	5.0
2	Upregulated genes in neutrophils compared to other blood cells		7.4E-13	3.8E-10	6.5
3	Downregulated in human embryonic stem cells *vs* differentiated counterparts		1.3E-13	5.8E-10	6.9
4	Downregulated genes in prostate cancer after androgen ablation therapy		4.9E-12	1.5E-8	20.5
5	SUZ12 target genes in human embryonic stem cells	X	5.2E-12	1.5E-8	6.4
6	Trimethylated H3K27 target genes in human embryonic stem cells	X	2.5E-11	4.8E-8	5.9
7	DrugBank targets - FDA approved		5.6E-11	1.0E-8	12.3
8	Upregulated genes in weakly invasive colon cancer cells		5.6E-11	1.0E-7	12.3
9	Polycomb group target genes in human embryonic stem cells	X	7.0E-10	8.4E-7	7.1
10	Upregulated genes in prostate cancer cells in response to synthetic androgen R1881		9.1E-10	9.5E-7	11.0
11	EED target genes in human embryonic fibroblasts	X	1.8E-9	1.8E-6	5.4
12	Trimethylated H3K27 target genes in human embryonic fibroblasts	X	3.4E-9	2.8E-6	3.7

PcG gain of function usually correlates with poor patient prognosis, and our results suggest that the NEARS preferentially contains many PcG targets. Thus, we sought to determine whether NEARS was also associated with clinical parameters indicative of aggressive PCa progression. Using the Oncomine database, we analyzed the differential expression of NEARS in tumors of different metastatic potential, grade, and prognosis. Strikingly, significant downregulation of NEARS was observed in metastatic compared to primary prostate malignancies in six independent datasets (Table 3, odds ratios = 2.4 to 6.0, *P* = 4.5 × 10^−4^ to 2.0 × 10^−26^). Likewise, NEARS underexpression was also recorded in eight clinical PCa datasets (Table 3, odds ratios = 2.2 to 7.6, *P* = 3.4 × 10^−4^ to 1.9 × 10^−8^). Finally, six additional datasets displayed reduced NEARS expression in patients with poor clinical outcome (Table 3, odds ratios = 2.4 to 15.2, *P* = 2.0 × 10^−3^ to 1.0 × 10^−11^), further supporting the idea that dowregulation of PcG target genes correlates with aggressive disease progression. Overall, our results demonstrating that NEARS contains many conserved PcG genomic targets and that multiple PcG genes are highly upregulated in NEPC strongly suggest that altered PcG-mediated silencing plays a key role in establishing and maintaining the NEPC phenotype.

**Table 3:** **Correlations between downregulation of NEARS and poor prognostic factors in clinical prostate tumors**

**Clinical parameters associated with NEARS**						
**Analysis**	**Concept**	**Dataset**	**Odds ratio**	***P*** **value**	**Percentile**	**Samples**
1° *vs* Met	Metastasis	Lapointe Prostate	2.4	1.3E-04	Top 10%	71
	Metastasis	LaTulippe Prostate	2.4	4.5E-04	Top 10%	32
	Metastasis	Yu Prostate	3.5	2.0E-07	Top 10%	88
	Metastasis	Grasso Prostate	3.8	5.6E-13	Top 10%	94
	Metastasis	Vanaja Prostate	4.6	8.4E-18	Top 10%	32
	Metastasis	Taylor Prostate 3	6.0	2.0E-26	Top 10%	150
Grade	Advanced Gleason Score	Wallace Prostate	2.2	3.4E-04	Top 10%	65
	Advanced Gleason Score	Vanaja Prostate	2.4	6.6E-06	Top 10%	27
	Advanced Gleason Score	Lapointe Prostate	3.4	1.8E-05	Top 5%	61
	Advanced Gleason Score	Tomlins Prostate	3.6	1.9E-08	Top 10%	30
	Advanced Gleason Score	Taylor Prostate 3	3.6	4.4E-08	Top 5%	130
	Advanced Gleason Score	Yu Prostate	4.4	4.1E-07	Top 5%	61
	Advanced Gleason Score	Setlur Prostate	7.6	2.8E-04	Top 1%	353
	High Grade	Bittner Prostate	2.5	3.3E-04	Top 5%	46
Outcome	Dead at 3 years	Setlur Prostate	2.4	2.0E-03	Top 10%	358
	Recurrence at 5 years	Taylor Prostate 3	3.1	1.3E-09	Top 10%	61
	Recurrence at 3 years	Taylor Prostate 3	3.5	1.0E-11	Top 10%	107
	Recurrence at 5 years	Nakagwa Prostate	10.1	1.0E-03	Top 10%	592
	Dead at 5 years	Setlur Prostate	10.7	2.9E-06	Top 1%	363
	Recurrence at 3 years	Nakagwa Prostate	15.2	7.9E-04	Top 5%	594

## Discussion

NEPC is an incurable malignancy which is expected to become more prevalent given the widespread use of potent AR-targeting drugs, which positively select for AR-negative NEPC cells [41]. However, the lack of suitable pre-clinical NEPC models has limited investigations into the molecular underpinnings of NEPC, therefore hampering therapeutic development of novel agents to treat this deadly disease. To circumvent this issue, we established a high fidelity, patient-derived xenograft model retaining classical NEPC features observed in the clinic [6] that allows us to investigate the molecular mechanisms involved in NEPC transdifferentiation. Using this model, we identified many PcG genes that are dysregulated in NEPC, a finding that was also observed in a clinical cohort and in additional patient-derived NEPC xenografts from the Living Tumor Laboratory. Moreover, we derived a NEARS that was predictive of PcG gain of function and aggressive disease progression. Based on these results, we propose that aberrant PcG-mediated silencing contributes to NEPC pathogenesis and that disrupting PcG activity may emerge as a valuable therapeutic strategy in NEPC.

Within the epigenetic landscape of NEPC, there was a global upregulation of repressive EpRs, and more than 70% of them were reported to directly interact with PcG complexes. Notably, all three genes encoding DNMTs were overexpressed in NEPC, suggesting that aberrant DNA methylation may synergize with alterations in PcG-mediated repression. In line with those results, increasing evidence supports the idea that PcG activity dictates DNMT recruitment to chromatin at target loci, implying a central role for PcG complexes in DNA methylation and its resulting epigenetic effects [42,43]. Aberrant DNA methylation has previously been reported in PCa, and the question of how DNA methylation patterns vary between PCa and NEPC remains unanswered [44]. Since cell fate transitions involve differential DNA methylation at enhancer regions [45], an attractive hypothesis is that PcG complexes and DNMTs synergize to regulate key enhancers relevant to neuroendocrine transdifferentiation. Consequently, future experiments should explore the distribution of DNA methylation in the context of genome-wide PRC1 and PRC2 chromatin binding in NEPC cells.

In line with the upregulation of PcG genes observed in NEPC, we derived a list of genes recurrently silenced in multiple NEPC models (NEARS) that was strongly associated with PcG-mediated repression. Moreover, silencing of NEARS in localized PCa tissue predicts an aggressive clinical course, consistent with the notion that certain PCas may be predisposed to acquire neuroendocrine-like features in a PcG-dependent manner. Interestingly, NEARS contained many PcG target genes in embryonic stem cells, implying that PcG activity in NEPC may suppress epithelial differentiation through silencing of genes specifying specialization into prostatic tissues, particularly since NEARS included some AR-regulated genes. In line with this idea, epigenetic reprogramming of a similar nature has been repeatedly observed in many aggressive tumor types featuring aberrant PcG-mediated repression [21]. In addition, neurogenesis is PcG-dependent [29,46], thus it seems likely that the activation of neuroendocrine-specific transcriptional programs may also be facilitated by increased PcG activity. Supporting this idea, we observed preferential overexpression of PcG genes CBX2 and EZH2 in SCLC, which represents a lung cancer subtype with neuroendocrine differentiation. Taken together, these results suggest that gene expression profiles regulated by PcG genes during normal embryogenesis are re-established in NEPC and possibly other neuroendocrine malignancies.

Despite playing imperative roles during embryonic development [47], CBX2 has been overlooked for many years in the cancer literature. Genetic inactivation of CBX2 (M33 in mice) causes lethality in 50% of subjects and the remaining progeny exhibit gonadal, adrenal, and splenic defects, reflecting a critical function for CBX2 in cellular differentiation [47,48]. In this paper, we report that CBX2 was consistently the most upregulated EpR in NEPC compared to PCa in our analyzed datasets. These findings support our recent discovery that CBX2 confers a genomic and transcriptomic profile consistent with that of an oncogene [49]. We have shown that high CBX2 expression correlates with poor patient outcome and more aggressive tumor phenotypes [49], in line with the clinical features of NEPC.

From a molecular standpoint, it is important to note that CBX2 upregulation observed in NEPC occurs in the context of overexpressed EZH2 and other PRC2 members, which likely alters the genomic distribution of the H3K27me3 mark. This has considerable mechanistic implications since CBX2 can directly bind H3K27me3 and recruit PRC1 to H3K27me3 sites, which solidifies transcriptional repression at target loci [24]. Thus, CBX2 overexpression might represent an alteration necessary to mediate the downstream epigenetic effect of PRC2 gain of function. Biochemical evidence supports this hypothesis since, although PRC1 composition varies in a context-dependent manner, PRC1 complexes found at H3K27me3 sites are preferentially enriched in CBX2 and not other CBX proteins [33]. While the relative contribution of CBX2 compared to other CBX members remains under investigation, the strong upregulation of CBX2 in NEPC, in addition to its critical role in cellular differentiation, suggest that CBX2 is functionally involved in the progression of NEPC.

Finally, we believe that the reported aberrations in PcG-mediated silencing have clear therapeutic implications, particularly given the emerging improvements in targeting the cancer epigenome [50,51]. In particular, we believe the interaction between CBX2 and H3K27me3 bridges the function of PRC2 and PRC1, thus representing a critical junction in this altered epigenetic pathway [52]. A few strategies can be put forward to therapeutically target this axis in the context of NEPC. First, small molecule inhibitors interfering with the methyltransferase activity of EZH2 have already been developed and warrant further investigation in NEPC [53]. Second, antagonists of the CBX2 chromodomain represent another promising path, as they would disrupt the binding between CBX2 and H3K27me3. At present, there are no small molecules directly targeting CBX2, although the development of CBX7 antagonists hints that a similar strategy could also be employed for CBX2 [54,55]. Third, antisense oligonucleotides (ASOs) may be used to reduce the expression of key PcG genes such as CBX2 and EZH2. An exponentially increasing number of ASOs have entered clinical testing, highlighting the potential of ASOs as therapeutic agents [56]. Taken together, our results highlight relevant alterations in Polycomb-mediated silencing that may be clinically targetable in lethal NEPC, thereby adding to the growing landscape of cancer epigenetics.

## Conclusions

Given the sparsity of adequate pre-clinical NEPC models, we investigated the epigenetic underpinnings of NEPC using innovative patient-derived xenograft models available at the Living Tumor Laboratory. Data obtained using this model was further validated in a clinical NEPC cohort. Using an integrative approach, we identified a striking upregulation of many key PcG genes, notably CBX2 and EZH2. In addition, we reported a clinically relevant dysregulation of PcG target genes in multiple NEPC models, consistent with a driving role for PcG complexes. Thus, our study reveals novel insights into NEPC pathogenesis and provides the rationale to establish therapeutic strategies aimed at disrupting altered PcG-mediated silencing.

## Methods

### Clinical expression datasets

Originating from the work of Beltran *et al*. the clinical NEPC cohort contained 7 NEPC tumors and 30 PCas which contained less than 10% stroma, as confirmed by a certified pathologist [7]. Prognostic analysis of NEARS, as well as the selected list of EpRs, was conducted using PCa datasets available from the Oncomine resource (www.oncomine.com) [31], which encompassed more than 3,800 patients. Clinical parameters assessed for differential gene expression included grade, metastasis, outcome, and stage. Analysis of literature-derived concepts correlated with NEARS was also done through the Oncomine resource, and the final list was unbiasedly determined using the lowest *P* values of associated concepts. Expression profiles of lung malignancies were generated by The Clinical Lung Cancer Genome Project (CLCGP) and Network Genomic Medicine (NGM) [37] (http://www.uni-koeln.de/med-fak/clcgp/).

### Gene lists

We established a list of targetable EpRs based on the following inclusion criteria: 1) being involved either in DNA methylation, histone acetylation, or histone methylation and 2) function as a writer, eraser, or reader of the epigenetic code. EpRs regulating DNA methylation were also subdivided into the same functional categories as those established for histone modifications (that is, writer = DNA methyltransferase (DNMT), eraser = ten eleven translocation (TET), reader = methyl CpG-binding domain (MBD)). Analysis of relevant literature [57-65] was conducted to identify such candidates, which were subsequently assessed in our NEPC datasets. In a similar way, a list of PcG genes was also derived from the literature using recent review papers written by authorities in the field [52,66,67]. We also derived a list of repressors that directly interact with PcG proteins based on literature findings (Additional file 3: Table S3). Finally, NEARS was established by combining the 185 genes downregulated in all three of the following datasets: 1) LTL331R/LTL331, 2) Clinical NEPC/PCa, and 3) LTL331/all LTL PCa [6].

### Immunohistochemistry

Establishment of paraffin-embedded tissue sections and immunostaining were conducted as previously described [6,68]. Detection was done using primary antibodies specific to PSA (rabbit polyclonal, Dako, Glostrup, Denmark), SYP (mouse monoclonal, Dako), CBX2 (rabbit polyclonal, Pierce, Rockford, USA), and EZH2 (rabbit monoclonal, Cell Signaling, Danvers, USA), as well as a goat anti-rabbit secondary antibody (Vector Laboratory, Peterborough, UK).

### Patient-derived xenografts

As previously described [6], the Living Tumor Lab (www.livingtumorlab.com) has established a bank of high-fidelity patient-derived xenografts. Tumor tissues were obtained from patients through a protocol approved by the Clinical Research Ethics Board of the University of British Columbia (UBC) and the BC Cancer Agency (BCCA). All patients signed a consent form approved by the Ethics Board (UBC Ethics Board #: H09-01628 and H04-60131; VCHRI #: V09-0320 and V07-0058). In this study, we used microarray data derived from ten PCa and three NEPC tumor lines, all of which retain the classical histological features of their respective subtype. The microarray gene expression data for these tumor lines have been previously deposited in the NCBI Gene Expression Omnibus (GEO) and are freely available under the accession number GSE41193.

### Statistical analysis

All statistical analyses were carried out with the Graphpad Prism software (version 6.0) using a statistical threshold of *P* ≤ 0.05 unless otherwise stated.

## Additional files

Additional file 1: Table S1. List of 147 targetable EpRs. List of 147 targetable EpRs and their associated fold changes in the clinical NEPC cohort and the 331R/331 model. Additional file 2: Table S2. Oncomine analysis revealing significant overexpression of the 22 selected EpRs. Oncomine analysis revealing significant overexpression of the 22 selected EpRs in metastatic compared to non-metastatic prostate cancer across multiple clinical datasets. Additional file 3: Table S3. Literature-reported direct interactions between PcG complexes and transcriptional repressors. Literature-reported direct interactions between PcG complexes and transcriptional repressors upregulated by at least 1.5-fold in both the clinical NEPC cohort and the 331R/331 model. Additional file 4: Table S4. List of investigated PcG genes. Known members of the PcG family and their associated polycomb repressive complex. Additional file 5: Table S5. Patient-derived xenograft models and their properties. Patient-derived xenograft models from Living Tumor Lab used for comparative NEPC/PCa analysis and their responses to androgen stimulation. Additional file 6: Figure S1. RB1 mRNA levels in patient-derived xenografts. Expression of CBX2, EZH2, and RB1 in tumor tissue derived from prostate adenocarcinoma or NEPC. Additional file 7: Table S6. Gene composition of NEARS. List of 185 overlapping genes significantly downregulated across multiple NEPC datasets.
