# Supplementary figures and images for: Polycomb-mediated silencing in neuroendocrine prostate cancer
Source: Clin Epigenetics. 2015 Apr 3;7(1):40. doi: 10.1186/s13148-015-0074-4 (PMC4391120; doi:10.1186/s13148-015-0074-4)

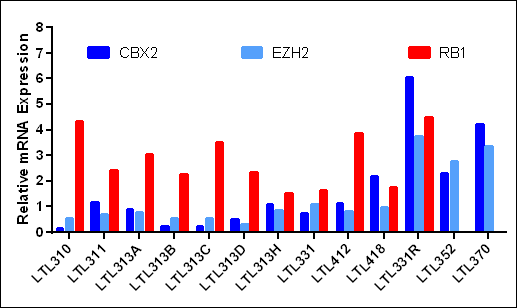

Supplement: Additional file 6: Figure S1. — RB1 mRNA levels in patient-derived xenografts. Expression of CBX2, EZH2, and RB1 in tumor tissue derived from prostate adenocarcinoma or NEPC. [file 13148_2015_74_MOESM6_ESM.tiff]
